# A Method to Estimate Sunshine Duration Using Cloud Classification Data from a Geostationary Meteorological Satellite (FY-2D) over the Heihe River Basin

**DOI:** 10.3390/s16111859

**Published:** 2016-11-04

**Authors:** Bingfang Wu, Shufu Liu, Weiwei Zhu, Mingzhao Yu, Nana Yan, Qiang Xing

**Affiliations:** Institute of Remote Sensing and Digital Earth (RADI), Chinese Academy of Sciences, Beijing 100094, China; liushf_263@163.com (S.L.); zhuww@radi.ac.cn (W.Z.); Yumz@radi.ac.cn (M.Y.); yannn@radi.ac.cn (N.Y.); xingqiang@radi.ac.cn (Q.X.)

**Keywords:** sunshine duration, cloud classification, FY-2D, Heihe River Basin

## Abstract

Sunshine duration is an important variable that is widely used in atmospheric energy balance studies, analysis of the thermal loadings on buildings, climate research, and the evaluation of agricultural resources. In most cases, it is calculated using an interpolation method based on regional-scale meteorological data from field stations. Accurate values in the field are difficult to obtain without ground measurements. In this paper, a satellite-based method to estimate sunshine duration is introduced and applied over the Heihe River Basin. This method is based on hourly cloud classification product data from the FY-2D geostationary meteorological satellite (FY-2D). A new index—FY-2D cloud type sunshine factor—is proposed, and the Shuffled Complex Evolution Algorithm (SCE-UA) was used to calibrate sunshine factors from different coverage types based on ground measurement data from the Heihe River Basin in 2007. The estimated sunshine duration from the proposed new algorithm was validated with ground observation data for 12 months in 2008, and the spatial distribution was compared with the results of an interpolation method over the Heihe River Basin. The study demonstrates that geostationary satellite data can be used to successfully estimate sunshine duration. Potential applications include climate research, energy balance studies, and global estimations of evapotranspiration.

## 1. Introduction

Sunshine duration, also known as the duration of real sunshine, is the time that the sun actually illuminates the Earth’s surface and is a measurement index of light resources. Sunshine duration is an important variable that is widely used in studies of atmospheric energy balance [1], analyses of the thermal loads on buildings, climate research, and the evaluation of agricultural resources [2,3] and is used to build estimation models of surface solar radiation [4,5,6]. Therefore, the accurate estimation of sunshine duration is important for researchers working in meteorology, hydrology, and agriculture.

The World Meteorological Organization (WMO) defines sunshine duration as the number of hours for which the direct solar irradiance is above 120 W/m^2^. There are many methods to measure sunshine duration, including direct measurement with sunshine duration recorders, the pyrheliometric method using direct irradiance from a pyrheliometer, and pyranometric algorithms using the global irradiance from a pyranometer [7,8,9]. Although these methods are accurate for measuring the sunshine duration at a station representing a certain area, using them for large regional assessments of sunshine duration is time-consuming and expensive because it requires numerous ground installations, especially when a large spatial coverage and high sampling frequency are desired. Interpolation methods with data from measurements at field meteorological stations are used to obtain the regional sunshine duration; however, the accuracy of interpolation methods is affected by the number and spatial distribution of meteorological stations. In addition, most meteorological stations are near cities, and there are areas without weather stations; as a result, the sunshine duration data from meteorological stations are often inadequate for representing the actual complex climate characteristics of geographic sunshine hours at a regional scale.

To obtain the sunshine duration at a regional scale, various empirical methods have been recommended. Two types of empirical methods are used to estimate sunshine duration in the field. One approach is the sunshine duration percentage method, including the astronomical sunshine percentage and the geographic sunshine percentage. The astronomical sunshine percentage is the ratio of sunshine duration to available sunshine duration, and the geographic sunshine percentage is the ratio of the actual sunshine duration to available sunshine duration. The astronomical sunshine percentage can be calculated using the sun trajectory equation [10]. The geographic sunshine percentage can be calculated by subtracting the amount of terrain masking from the astronomical sunshine percentage [11,12]. The sunshine duration can then be calculated using the Angstrom method based on the assimilation of radiation data [13,14,15,16,17,18,19,20,21], but this empirical method overly relies on the accuracy of the assimilation of radiation data. The other approach is based on clearness index, the amount of clouds, total cloud cover, precipitation, wind speed, air pollution index (API), hourly or shorter time interval cloud-type, and surface incoming direct radiation methods, in which several formalized statistical models have been studied. Empirical equations have been proposed based on the relative sunshine duration and the aforementioned readily available data from ground measurements (meteorological, air quality and radiometric data from various stations) and remote sensing data [7,22,23,24]. Consequently, this empirical method does not consider the changes in the amount of clouds, air pollution index, and precipitation and can calculate only the monthly sunshine duration, or the cloud type data between sunrise and sunset. Surface incoming direct radiation data must be required at the same time in some empirical methods [24,25,26], and it cannot accurately estimate the daily sunshine duration on the premise that only the cloud type data—not surface incoming direct radiation data—can be used.

With the development of satellite remote sensing technology, it is possible to continuously observe a wide range of cloud cover at a regional scale. In particular, geostationary meteorological satellites can provide information about the types of cloud per hour or shorter time interval, and different cloud types will impact the solar radiation, which will ultimately affect the daily sunshine duration. Therefore, hourly or shorter time interval geostationary meteorological satellite cloud classification data can be used directly to calculate sunshine duration without the need for additional data.

The purpose of our paper is to investigate a method to derive the sunshine duration only from geostationary meteorological satellite cloud classification data, and a new index—the cloud type sunshine factor—is proposed, which can reflect the influence of the hourly cloud type on solar radiation. To accurately estimate the sunshine duration, the Shuffled Complex Evolution Algorithm (SCE-UA) was used to calibrate different cloud types of sunshine factors based on ground measurement data. The estimated sunshine duration values from this proposed new algorithm were validated with independent ground observation data, and the spatial distribution was compared with the results of interpolation methods.

## 2. Materials and Methods

### 2.1. Study Site and Datasets

**Figure 1:**
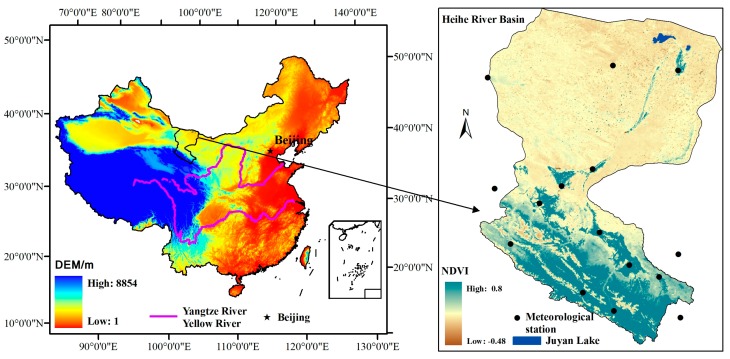
Location of observation sites in the Heihe River Basin.

The Heihe River Basin (~128,900 km^2^), the second largest inland river basin in the country, is located in the arid northwestern region of China between 97°24′–102°10′ E and 37°41′–42°42′ N. Its elevation ranges from approximately 5000 m in the upper reaches to 1000 m downstream. The river originates in the Qilian Mountains and flows through the Hexi corridor of the province of Gansu from the Yingluo Gorge, through the Zhengyi Gorge, and then northward into the Ejina oasis in the western part of the Inner-Mongolia Plateau before finally discharging into the eastern and western Juyan Lakes (Figure 1). The landscape varies from glaciers and frozen soil to alpine meadow, forest, irrigated cropland, riparian ecosystem, bare gobi, and desert. The highest air temperatures are approximately 40 °C in downstream areas in the summer, and the lowest fall to approximately −40 °C in the upper watershed in the winter. The mean annual rainfall across the basin is 110.9 mm·yr^−1^ (1980–2010), and the annual precipitation in the upstream area is more than 350 mm·yr^−1^; it is 100–250 mm·yr^−1^ in the middle reaches, and the annual precipitation in the downstream area is less than 50 mm·yr^−1^.

The high heterogeneity of the underlying surface and the strong seasonal weather changes in the Heihe River Basin can better test the feasibility of the new method proposed in this paper.

### 2.2. Methodology

#### 2.2.1. Data and Pre-Processing Methods

##### Sunshine Duration Observation Data

Data from 14 meteorological stations covering the Heihe River Basin and its surrounding areas were used (Table 1) and distributed in different land use areas of the mountain and plains areas. Each station is equipped with an observation system that records 6 meteorological variables. Diurnal meteorological data include the sunshine duration, air temperature, air pressure, air humidity, wind speed, and rainfall. The meteorological data were provided by the Chinese National Meteorological Bureau, and the sunshine duration data used in the study were from 2007 to 2008 and can be downloaded from http://cdc.cma.gov.cn/cdc_en/home.dd. Quality control of the data was performed by the suppliers.

##### Geostationary Meteorological Cloud Products Data

Cloud product data in each hour covering the Heihe River Basin from 2007 to 2008 were obtained (17,544 images) from the Chinese Meteorological Administration’s (CMA) data services website in HDF format. A geographic lookup table (GLT) file downloaded from the National Satellite Meteorological Center was used to obtain the cloud classification data in a geographic projection for the target area and the cloud classification coverage type (cloud type) [27], ranging from 1 to 7 and in the order of “Number” in Table 2. These data were generated by the FengYun-2D (FY-2D) satellite, the second operational vehicle of the first-generation geostationary meteorological satellite system operated by the CMA. The satellite was launched on 8 December 2006 and is located above the Equator at longitude 86.5° E at an altitude of 35,800 km. Its objective is to monitor clouds and temperatures above China and neighbouring areas of the Asia-Pacific region. The upgraded Stretched-Visible and Infrared Spin-Scan Radiometer (S-VISSR) is one of the major payloads on board FY-2D. This optical imaging radiometer consists of one visible channel with a resolution of 1.25 km and four infrared channels with resolutions of 5 km at different wavelengths, including VIR: 0.55–0.90 μm; IR1: 10.3–11.3 μm; IR2: 11.5–12.5 μm; IR3: 6.3–7.6 μm; and IR4: 3.5–4.0 μm (Xu, 2010a, 2010b). The radiometer can acquire one image covering the Earth’s surface from 60° N to 60° S and from 45° E to 165° E per hour.

#### 2.2.2. Modelling Sunshine Duration

The influence of different cloud types on solar radiation is mainly reflected in the fact that the diurnal variation of cloud types leads to a variation of hourly illumination intensity, which then affects the daily sunshine duration. Therefore, we propose a new index—the cloud type sunshine factor (SF)—to characterize the magnitude of the effects of different cloud types of Fengyun (FY) geostationary meteorological satellite on the sunshine duration per hour over the Heihe River Basin. This new sunshine factor was combined with the hourly cloud classification data between sunrise and sunset to estimate the sunshine duration (Equation (1)). The sunrise time and sunset time over the Heihe River Basin can be calculated from the geographic latitude and earth declination based on the day of the year:

(1) $$FY_{\mathrm{sunt}}=\sum_{i=h_{\mathrm{sr}}+0.25}^{i=h_{\mathrm{ss}}-0.25}SF_{i}\times T_{\mathrm{gap}}$$

where FY_sunt_ is the sunshine duration (between 15 min (+0.25 h) after the start of sunrise and 15 min (−0.25 h) before sunset and the accumulation of sunshine factors); SF is the FY-2D cloud type sunshine factor, which is the index for the hourly FY-2D hourly cloud type data from sunrise to sunset; T_gap_ is an hour’s interval with a value of 1; h_sr_ and h_ss_ are the times of sunrise and sunset, respectively; i is a time series that ranges between sunrise and sunset at the local time.

##### FY-2D Cloud Type Sunshine Factor

Under the condition that the correlation coefficient is greater than or equal to 0.9, based on Equation (1), the FY-2D cloud type sunshine factors (FY-2D-SF) at each meteorological station were fitted and combined with the hourly FY-2D cloud type data from sunrise to sunset and the measured sunshine duration data of each meteorological station at the different dates over the Heihe River Basin in 2007. Table 3 shows the range of the FY-2D-SF, which indicates the range of cloud type sunshine factors present high fluctuations at the regional scale. According to the data, the FY-2D-SF of CLS was the highest in 2007, from 0.78 to 0.98. The second highest was the CIS, from 0.45 to 0.58. The third was STA, from 0.30 to 0.41. MIP, ALN, and CIRS were relatively equal. The lowest FY-2D-SF appeared in the CUC.

##### FY-2D Cloud Type Sunshine Factor Estimation

In the previous section, the different cloud types corresponding to the sunshine factor range over the different meteorological stations were established. Next, we used an optimization algorithm to calibrate different cloud type sunshine factors at the regional scale. In this paper, the Shuffled Complex Evolution Algorithm (SCE-UA) based on the simple algorithm was used to optimize the FY-2D cloud typed sunshine factor over the Heihe River Basin for 2007, which is an effective method for solving nonlinear constrained optimization problems and can be used to find a global optimal solution. The algorithm was originally applied to a hydrological model, and we applied it to the optimization of FY-2D cloud type sunshine factors in this paper [28].

#### 2.2.3. Model Performance Assessment

The performances of the proposed methods for estimating sunshine duration were assessed based on widely used goodness-of-fit statistics [29], including the coefficient of determination (R^2^), mean absolute error (MAE), root mean square error (RMSE), and index of agreement (d). These parameters are defined as follows:

(2) $$R^{2}=\frac{{\left[\sum_{i=1}^{n}\left(O_{i}-\bar{O}\right)\left(P_{i}-\bar{P}\right)\right]}^{2}}{\left[\sum_{i=1}^{n}{\left(O_{i}-\bar{O}\right)}^{2}\sum_{i=1}^{n}{\left(P_{i}-\bar{P}\right)}^{2}\right]}$$

(3) $$\mathrm{MAE}=n^{-1}\sum_{i=1}^{n}\left|P_{i}-O_{i}\right|$$

(4) $$\mathrm{RMSE}=\sqrt{n^{-1}\sum_{i=1}^{n}{\left(P_{i}-O_{i}\right)}^{2}}$$

(5) $$d=1-\frac{\sum_{i=1}^{n}{\left(P_{i}-O_{i}\right)}^{2}}{\sum_{i=1}^{n}{\left(\left|P_{i}-\bar{O}\right|+\left|O_{i}-\bar{O}\right|\right)}^{2}}$$

In the above equations, Q_i_ is the actual measurement, P_i_ is its estimate, $\bar{O}$ is the mean measurement, $\bar{P}$ is the mean of the estimates, and n is the sample size. Colaizzi and Liu suggest Equation (1) that a model performs well when the MAE is less than 50% of the measured standard deviation, Equation (2) that there are few outliers when the RMSE is not greater than 50% of the MAE, and Equation (3) that the higher the value of d, the better the model performance [30,31].

## 3. Results

Table 4 shows the final FY-2D cloud type sunshine factor over the Heihe River Basin after the application of the optimization algorithm. The sunshine factors of CLS, MIP, ALN, CIS, CIRS, CUC, and STA were 0.9, 0.21, 0.25, 0.51, 0.24, 0.13, and 0.35, respectively.

**Figure 2:**
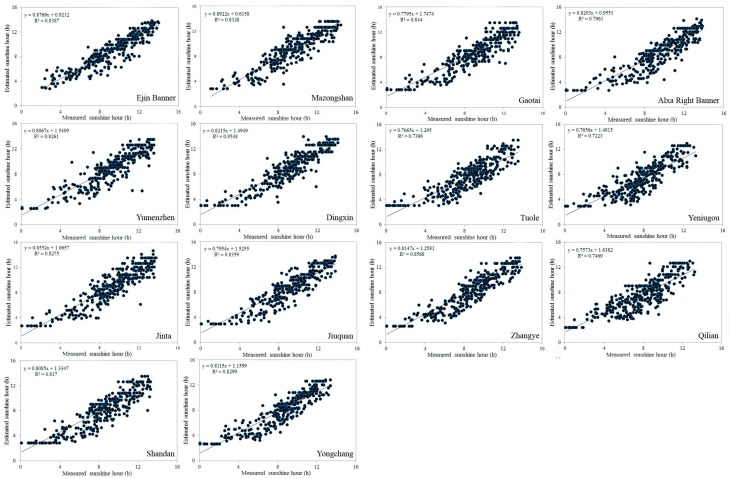
Comparison of the measured sunshine duration and estimated sunshine duration the different meteorological station over the Heihe River Basin in 2008.

Based on these sunshine factor results, the daily sunshine duration values were calculated over the Heihe River Basin in 2008, and Table 5 and Figure 2 show the validation results of the estimation of the daily sunshine duration and ground measurement sunshine duration data at each station in 2008. For the whole Heihe River Basin, the coefficients of determination (R^2^) were greater than 0.89, except for three stations in the mountains (Tuole, Yeniugou, and Qilian). There was therefore a strong correlation between the actual sunshine duration and the sunshine duration estimated using the new method. The lower coefficients of determination in the mountain stations (Tuole, Yeniugou, and Qilian) are mainly due to the effects of topography; where topographic effects reduce the values of direct solar irradiance to below 120 W/m^2^, there will be no registration of the sunshine duration. The d values of all stations were greater than 0.990, again suggesting good performance. The difference between the RMSE and MAE was less than 40% of the MAE, meaning there are few outliers in the estimated ET values, according to Colaizzi.

**Figure 3:**
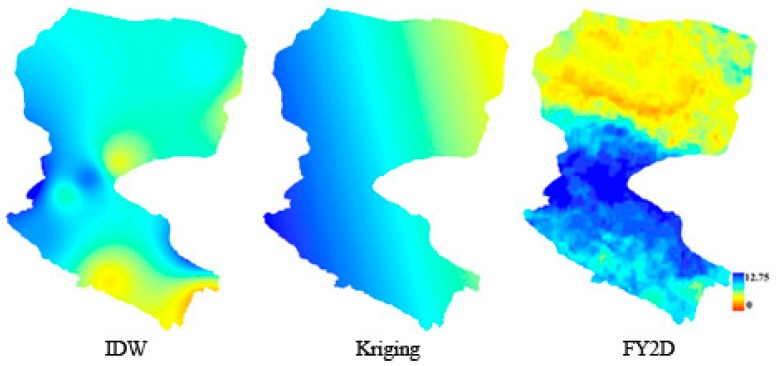
Spatial distribution of sunshine duration distribution estimated by IDW (**left**), Kriging (**middle**), and the proposed model (**right**) over the Heihe River Basin on 16 July 2008.

Figure 3 shows that the spatially distributed sunshine duration on the left map was obtained via IDW spatial interpolation (inverse distance weighting with exponent 2), and the middle map was obtained by the Kriging method (ordinary and exponential). At the same time, the different methods (ordinary or universal) were chose and different semivariogram models (e.g., spherical, circular, Gaussian, and linear) were used in the Kriging interpolation. The results from the Kriging interpolation was used as baseline data for comparison among the IDW method results and the proposed method results. Thus, the comparison results of the three methods show that it was large differences for the spatial distribution pattern.

Because the ground measurements are sparsely distributed in the Heihe River Basin, especially in the downstream regions, regardless of which interpolation method was used for the interpolation of sunshine duration, the result from different interpolation methods and the method proposed in this paper differs in spatial distribution. Moreover, Figure 2 shows that the method proposed here has a higher accuracy in the Heihe River Basin compared with the measured sunshine duration from different stations; therefore, it is clear that the proposed method is better able to show the spatial distribution variation of the sunshine duration.

## 4. Discussion

In this paper, we demonstrate a method to derive the sunshine duration using only the cloud classification data from a geostationary meteorological satellite (FY-2D) without depending on continuous ground measurements. This method uses cloud classification data to calibrate the FY-2D cloud type sunshine factor and is combined with the sunrise and sunset to estimate the daily sunshine duration. High correlations were obtained between the estimated and measured in situ sunshine duration at different ground measurement stations, and the method accurately portrays the variation in the spatial distribution of the sunshine duration over the Heihe River Basin. This new method has the potential to estimate the diurnal variation of surface net radiation and evapotranspiration over large regions.

Remotely sensed data describe the pixel sunshine duration. For cloud classification data from FY-2D, the pixel value represents the average sunshine duration for a 5 km × 5 km area. In addition, some of the smaller clouds in the cloud classification data have not been accounted for, which can affect the sunshine duration estimated by the method proposed in this paper. Ground measurements of sunshine duration represent a much smaller area, which does not match with the area covered by a pixel. Consequently, some outliers can be expected in Table 5 and Figure 2, in which the estimated values are compared with the measured values. However, most meteorological stations in the plain areas are near cities, and the terrain around them is relatively flat and has a relatively homogeneous surface coverage. Thus, the ground measurement values in the plains areas can be assumed to represent the average value for the surrounding region and can be appropriately matched with remote sensing pixel values. In contrast, in the mountains, due to the effects of topography and the heterogeneity of the mountain surface, some areas have low direct solar irradiance, which leads to a lower correlation between the actual sunshine duration and the estimated sunshine duration, as shown in the results in the Tuole, Yeniugou, and Qilian stations.

The proposed method is an empirical method, and the sunshine factors of different cloud types in the Heihe River Basin were calculated from meteorological station data. The sunshine factors must be calibrated using the local ground measurements of sunshine duration if the proposed method is applied in other regions.

There is a ceiling value of the sunshine duration at a specific geographical latitude independent of the cloud type. For example, the sunshine duration is always zero in the polar night in the winter in the Arctic. The sunshine hours will also be affected by the topography. In addition, different cloud amounts have an impact on the sunshine factor even with the same cloud types. Further study will be carried out to take these factors into account, and the results may improve the quality of operational sunshine duration data.

## 5. Conclusions

A method was developed to estimate sunshine duration using cloud classification data from a geostationary meteorological satellite. The method was calibrated and validated in the Heihe River Basin of China. The method uses hourly cloud type data from the Fengyun-2D geostationary meteorological cloud products and data from the field measurements of sunshine duration to estimate a new index—the FY-2D cloud type sunshine factor. Using a wide range of meteorological stations within the Heihe River Basin in 2007, the Shuffled Complex Evolution Algorithm (SCE-UA) was used to optimize the FY-2D sunshine factor over the Heihe River Basin. This new sunshine factor combined with the hourly cloud classification data between sunrise and sunset can be used to estimate sunshine duration, and high correlations were obtained between the estimated and measured sunshine hours at different stations in 2008, with coefficients of determination (R^2^) greater than 0.89, except for three stations in the mountains. The spatial distribution analysis of the sunshine duration showed that its spatial distribution was better represented by the new method than by a method based on interpolation between meteorological stations.

## Figures and Tables

**Table 1:** Station names, numbers, and coordinates.

Station		Longitude E (°)	Latitude N (°)	Elevation (m)
No.	Name			
52267	Ejin Banner	101.09	41.94	940.50
52323	Mazongshan	97.11	41.51	1770.40
52436	Yumenzhen	97.55	39.84	1526.00
52446	Dingxin	99.51	40.31	1177.40
52447	Jinta	98.91	40.00	1270.50
52533	Jiuquan	98.50	39.70	1477.20
52546	Gaotai	99.79	39.36	1332.20
52576	Alxa Right Banner	101.43	39.14	1510.10
52633	Tuole	98.01	39.03	3367.00
52645	Yeniugou	99.58	38.42	3320.00
52652	Zhangye	100.46	38.91	1482.70
52657	Qilian	100.24	38.19	2787.40
52661	Shandan	101.08	38.77	1764.60
52674	Yongchang	101.58	38.18	1976.90

**Table 2:** FengYun-2D (FY-2D) cloud classification codes and cloud type.

Number	Code	Cloud Type	Abbreviation
1	0/1	Clear Sky	CLS
2	11	Mixed pixels	MIP
3	12	Altostratus or nimbostratus	ALN
4	13	Cirrostratus	CIS
5	14	Cirrus spissatus	CIRS
6	15	Cumulonimbus	CUC
7	21	Stratocumulus or altocumulus	STA

**Table 3:** The range of the FY-2D cloud type sunshine factors (FY-2D-SF) in different meteorological stations.

Station Name	CLS	MIP	ALN	CIS	CIRS	CUC	STA
Ejin Banner	0.86–0.93	0.18–0.25	0.22–0.28	0.44–0.56	0.18–0.26	0.10–0.15	0.30–0.39
Mazongshan	0.80–0.90	0.16–0.29	0.20–0.27	0.47–0.54	0.19–0.26	0.12–0.14	0.32–0.36
Yumenzhen	0.83–0.96	0.18–0.27	0.24–0.29	0.48–0.55	0.17–0.27	0.09–0.14	0.30–0.41
Dingxin	0.79–0.98	0.18–0.26	0.21–0.28	0.50–0.54	0.22–0.27	0.08–0.15	0.32–0.38
Jinta	0.81–0.94	0.20–0.24	0.25–0.26	0.46–0.57	0.18–0.27	0.10–0.15	0.33–0.37
Jiuquan	0.80–0.94	0.17–0.28	0.22–0.27	0.46–0.55	0.23–0.25	0.09–0.16	0.31–0.36
Gaotai	0.85–0.95	0.18–0.27	0.20–0.29	0.49–0.53	0.22–0.28	0.10–0.14	0.30–0.37
Alxa Right Banner	0.82–0.93	0.18–0.27	0.23–0.26	0.48–0.58	0.18–0.27	0.11–0.15	0.32–0.36
Tuole	0.80–0.96	0.20–0.25	0.21–0.26	0.45–0.55	0.23–0.26	0.10–0.15	0.34–0.37
Yeniugou	0.80–0.93	0.18–0.27	0.23–0.28	0.47–0.56	0.19–0.25	0.12–0.14	0.30–0.38
Zhangye	0.87–0.92	0.15–0.22	0.22–0.32	0.48–0.58	0.21–0.25	0.09–0.15	0.33–0.36
Qilian	0.78–0.92	0.20–0.25	0.19–0.29	0.49–0.52	0.18–0.27	0.11–0.14	0.31–0.38
Shandan	0.83–0.96	0.18–0.28	0.22–0.28	0.47–0.53	0.22–0.25	0.10–0.16	0.32–0.37
Yongchang	0.81–0.95	0.18–0.27	0.20–0.30	0.48–0.53	0.20–0.25	0.12–0.15	0.34–0.36
Range	0.78–0.98	0.15–0.29	0.19–0.32	0.45–0.58	0.17–0.28	0.08–0.16	0.30–0.41

**Table 4:** FY-2D cloud type sunshine factor over the Heihe River Basin.

Code	Surface Cloud Coverage Type (Cloud Classification)	Sunshine Factor
0/1	Clear sky	0.9
11	Mixed pixels	0.21
12	Altostratus or nimbostratus	0.25
13	Cirrostratus	0.51
14	Cirrus spissatus	0.24
15	Cumulonimbus cloud	0.13
21	Stratocumulus or altocumulus	0.35

**Table 5:** Validation results of the estimation of daily sunshine duration over the Heihe River Basin.

Station Name	Observed Average	Estimated Average	R^2^	MAE	RMSE	d
Ejin Banner	9.175	8.949	0.916	0.784	1.105	0.997
Mazongshan	9.668	9.241	0.913	0.914	1.220	0.996
Yumenzhen	8.899	8.720	0.909	1.048	1.418	0.994
Dingxin	9.148	9.010	0.925	0.951	1.269	0.996
Jinta	9.293	9.013	0.910	1.035	1.332	0.995
Jiuquan	8.554	8.330	0.914	1.105	1.425	0.994
Gaotai	8.317	8.231	0.919	1.137	1.462	0.993
Alxa Right Banner	9.395	8.662	0.892	1.184	1.558	0.993
Tuole	8.352	7.697	0.860	1.296	1.626	0.991
Yeniugou	7.470	7.200	0.850	1.235	1.551	0.990
Zhangye	8.280	7.999	0.925	1.099	1.397	0.990
Qilian	7.481	7.481	0.864	1.307	1.613	0.990
Shandan	8.045	7.838	0.904	1.136	1.463	0.993
Yongchang	8.264	7.866	0.911	1.084	1.379	0.994
